# Racial and ethnic differences in white matter hypointensities: The role of vascular risk factors

**DOI:** 10.1002/alz.70105

**Published:** 2025-03-27

**Authors:** Farooq Kamal, Roqaie Moqadam, Cassandra Morrison, Mahsa Dadar

**Affiliations:** ^1^ Department of Psychiatry McGill University Montreal Quebec Canada; ^2^ Douglas Mental Health University Institute Verdun Quebec Canada; ^3^ Department of Psychology Carleton University Ottawa Ontario Canada

**Keywords:** cognitive aging, dementia, ethnicity, race, vascular risk factors, white matter hypointensity

## Abstract

**INTRODUCTION:**

White matter hypointensities (WMHs) are markers of cerebrovascular pathology associated with cognitive decline. Reports of racial and ethnic differences in WMHs have been inconsistent across studies. This study examined whether race and ethnicity influence WMH burden and whether vascular risk factors explain these differences.

**METHODS:**

Data from the National Alzheimer's Coordinating Center included 7132 Whites, 892 Blacks, 283 Asians, and 661 Hispanics. Baseline and longitudinal WMHs were examined using linear regression and mixed‐effects models across racial and ethnic groups, controlling for demographics and vascular risk factors.

**RESULTS:**

Adjusting for vascular risk factors reduced WMH burden differences and eliminated differences in temporal regions in Black versus White older adults. For Hispanics, differences became significant after adjusting for vascular risk factors.

**DISCUSSION:**

Although some racial and ethnic WMH disparities are influenced by vascular risk factors, others persist, highlighting the need for multidimensional approaches when targeting WMHs in diverse populations.

**Highlights:**

- Current research is inconsistent as to whether there are racial differences in white matter hypointensities (WMHs).
- Blacks exhibit higher WMH burden than Whites, mediated by vascular factors.
- In Hispanics, WMH differences emerged only after adjusting for vascular risk factors.

## 1 BACKGROUND

One of the critical pathological markers observed in the aging brain is damage to white matter due to cerebral small vessel disease (CSVD). These changes are often observed as white matter hyperintensities^1^ on T2‐weighted (T2w) or fluid‐attenuated inversion recovery (FLAIR) magnetic resonance imaging (MRI), and as white matter lesions or white matter hypointensities (WMHs) on T1w MRI. WMHs are hallmarks of CSVD, which present in cognitively healthy older adults, but are also associated with an increased risk of cognitive decline, progression to mild cognitive impairment (MCI), and dementia.^2^, ^3^, ^4^, ^5^ Higher WMH burden correlates with future cognitive impairment in both aging and neurodegenerative disorders,^6^, ^7^, ^8^, ^9^, ^10^, ^11^ further emphasizing that WMHs are important indicators of cerebrovascular health and neurodegenerative disease risk.

Vascular risk factors including hypertension, diabetes, and elevated body mass index (BMI) are contributors to WMH burden and independently increase the risk of dementia.^12^, ^13^ These risk factors are also disproportionately prevalent in diverse populations, particularly among Blacks. The increased risk factor prevalence may contribute to racial disparities observed in WMH burden,^14^, ^15^, ^16^ and increased dementia risk among Black compared to White older adults.^17^, ^18^ Targeting these factors may thus reduce the heightened risk of dementia in Black individuals, who are nearly twice as likely as White individuals to develop dementia.^19^

Nonetheless, the evidence for WMH burden differences between racial and ethnic groups remains inconsistent. Some studies report no significant differences in WMH burden between Black and White individuals,^20^, ^21^ whereas others indicate higher WMH levels in Blacks compared to Whites.^14^, ^16^, ^22^, ^23^, ^24^ For example, Divers et al.^21^ found comparable WMHs between Black and White individuals; however, when controlling for vascular risk factors, others^14^, ^25^, ^26^ report that many of the racial group differences were no longer significant, indicating that vascular risk factors contribute to race differences in WMH burden. Similarly, for Hispanic populations, they exhibit more severe WMH burden compared to Whites, with these differences being partially attributable to differences in vascular disease.^14^ On the other hand, Asian populations may not differ from White populations in terms of WMH burden.^26^ A recent systematic review found that current studies on WMH burden in aging include mainly White participants (55%), followed by 30% Black, 13.9% Hispanic, 0.6% Asian, and 0.5% Other.^27^ The limited research on these samples highlights the need for more diverse datasets to clarify the role of race and ethnicity in WMHs.

**RESEARCH‐IN‐CONTEXT**

1. **Systematic review**: The authors reviewed the literature on racial and ethnic differences in white matter hypointensities (WMHs), using traditional sources such as PubMed, focusing on studies that examined baseline and longitudinal WMH burden. Although previous aging studies have highlighted racial differences in WMH burden, the impact of vascular risk factors are inconsistent.
2. **Interpretation**: Our findings suggest that racial differences in WMH burden between Black and White older adults are influenced by vascular risk factors. However, differences in Hispanic populations emerged only after adjusting for these factors. These results suggest that racial WMH differences are mediated by vascular factors and that ethnic WMH differences may be mediated by non‐vascular factors.
3. **Future directions**: Future research should investigate non‐vascular factors, such as chronic stress with comprehensive vascular measures. Longitudinal studies with diverse samples are needed to confirm these findings and assess regional WMH progression across older adults.

Our previous study examined the influence of vascular risk factors on WMH differences between White and Black individuals.^25^ This study observed that Black participants had higher baseline WMH volume in frontal, parietal, and deep brain regions compared to White older adults. However, after controlling for vascular risk factors, most of these racial group differences were no longer significant, except for longitudinal increases in parietal WMH volume. These findings suggest that the greater prevalence of vascular risk factors among Black compared to White older adults contributes to some differences in WMHs. Although these findings were informative, the relatively small sample size of non‐White individuals, which primarily included Black individuals, limited the generalizability of the results to other racial groups, such as Asian individuals, and did not explore the potential influence of ethnicity on the outcomes.

To further our understanding of how race and ethnicity affect WMH burden and the influence of vascular risk factors, investigation in a larger, more diverse dataset, particularly one with representation of racial and ethnic groups was needed. The National Alzheimer's Coordinating Center (NACC) dataset, which includes clinical, MRI, and neuropsychological data from diverse participants, was utilized to explore WMH differences between races and ethnicities. Using the NACC dataset provides an opportunity to examine both overall and regional WMH differences with and without controlling for vascular risk factors. Regional WMH measures, especially in the parietal and frontal lobes, may be particularly relevant, given their association with Alzheimer's disease (AD) and small vessel disease.^28^, ^29^, ^30^ By validating these findings in a broader population, this study aims to further clarify how vascular health impacts racial and ethnic disparities in WMH burden.

## 2 METHODS

### 2.1 National Alzheimer's Coordinating Center

Longitudinal and baseline data was obtained from the National Alzheimer's Coordinating Center (NACC, https://naccdata.org/) database, including the NACC Uniform Data Set (UDS) and MRI Data Set.^31^, ^32^, ^33^ Two subsets of participants were selected from the NACC dataset for this study: (1) if they had clinical data and visual WMH assessments available, and (2) if they had additional MRI scans available to extract regional WMH measures. Demographic details for the full clinical dataset are presented in Table 1, and details for the MRI subset are presented in Table 2. WMH burden in the clinical dataset was classified into three levels (i.e., low, moderate, and extensive) based on the IMAGMWMH and IMAGEWMH variable names, as recorded by clinicians and provided by the NACC. We utilized the Cardiovascular Health Study (CHS) criteria for classifying WMH severity in the NACC dataset, where WMHs are recorded as moderate (under IMAGMWMH category; CHS score 5–6) or extensive (under IMAGEWMH; CHS score 7–8). This approach is based on clinician assessments using the CHS criteria, a well‐established and widely validated approach for evaluating WMH severity,^34^ and has been used in previous research studies.^35^, ^36^ For this longitudinal dataset, there were a total of 8307 non‐Hispanic participants comprising of 7132 White participants (with 13,333 timepoints), 892 Black participants (with 1524 timepoints), and 283 Asian participants (with 557 timepoints) with completed race information. When examining ethnicity, the clinical dataset comprised a total of 8968 participants consisting of 8307 non‐Hispanic participants (15,406 timepoints) and 661 Hispanic participants (1289 timepoints) with completed information.

**TABLE 1:** Descriptive information for vascular risk factors in the clinical dataset.

	White (*n* = 7132)	Black (*n* = 892)	Asian (*n* = 283)	Hispanic (*n* = 661)	non‐Hispanic (*n* = 8307)
Age, mean ± SD	71.36 ± 9.23	72.26 ± 8.82^a^	71.27 ± 9.53	71.47 ± 8.66	71.38 ± 9.19
Education, mean ± SD	16.31 ± 2.67	15.08 ± 2.81^a^	16.48 ± 3.13	13.61 ± 4.54	16.18 ± 2.73^c^
BMI, mean ± SD	26.72 ± 5.00	29.13 ± 6.04^a^	23.90 ± 3.73^b^	28.09 ± 5.04	26.89 ± 5.17^c^
Male, *n* (%)	3364 (47%)	244 (27%)^a^	116 (41%)^b^	236 (37%)	3724 (45%)^c^
Diagnosis, *n* (%)					
NC	3869 (54%)	549 (62%)	167 (59%)	301 (47%)	4330 (52%)
MCI	656 (9%)	95 (11%)	28 (10%)	130 (20%)	837 (10%)
AD	2607 (37%)	248 (28%)	88 (31%)	230 (36%)	3140 (38%)
Hypertension, *n* (%)	2990 (42%)	625 (70%)^a^	131 (46%)	374 (59%)	3740 (45%)^c^
Diabetes, *n* (%)	665 (9%)	239 (27%)^a^	50 (18%)^b^	167 (25%)	948 (11%)^c^

*Note*: Age, education, and BMI are reported as the mean ± SD, whereas sex, diagnostic status, hypertension, and diabetes are presented as the total number of participants and the percentage of the sample for each group.

Abbreviations: AD, Alzheimer's disease, BMI, body mass index; MCI, mild cognitive impairment; NC, normal control; SD, standard deviation.

^a^ Represents statistically significant racial group differences. Black participants had significantly lower education levels, a lower proportion of male participants, higher rates of diabetes and hypertension, and higher BMI compared to White participants.

^b^ Represents statistically significant racial group differences. Asian participants had significantly lower BMI, lower proportion of male participants, and higher rate of diabetes levels compared to White participants.

^c^ Represents statistically significant ethnic group differences. Hispanic participants had significantly lower education levels, higher BMI, and higher rates of diabetes and hypertension compared to non‐Hispanic participants.

**TABLE 2:** Descriptive information for vascular risk factors in the MRI dataset.

	White (*n* = 1876)	Black (*n* = 260)	Asian (*n* = 55)	Hispanic (*n* = 207)	non‐Hispanic (*n* = 2191)
Age, mean ± SD	70.38 ± 9.06	70.98 ± 9.12	74.80 ± 9.60^b^	72.32 ± 7.76	70.56 ± 9.11^c^
Education, mean ± SD	15.93 ± 2.85	14.53 ± 3.09^a^	15.62 ± 3.03	11.25 ± 4.88	15.76 ± 2.92^c^
BMI, mean ± SD	26.77 ± 4.71	29.73 ± 5.49^a^	23.62 ± 2.95^b^	28.93 ± 5.33	27.04 ± 4.90^c^
Male, *n* (%)	869 (46%)	75 (29%)^a^	19 (35%)	66 (32%)	1228 (56%)^c^
Diagnosis, *n* (%)					
NC	1098 (59%)	181 (70%)	30 (55%)	131 (63%)	1309 (60%)
MCI	176 (9%)	37 (14%)	8 (15%)	17 (8%)	221 (10%)
AD	602 (32%)	42 (16%)	17 (31%)	59 (29%)	661 (30%)
Hypertension, *n* (%)	779 (42%)	181 (70%)	31 (56%)^b^	129 (62%)	991 (45%)^c^
Diabetes, *n* (%)	180 (10%)	94 (36%)^a^	14 (25%)^b^	61(29%)	288 (13%)^c^
Frontal WMH	263–80447	279–66939	493–14448	481–28876	263–80447
Parietal WMH	4–47906	29–36867	42–11338	11–19586	4–47906
Temporal WMH	1–7643	7–5492	18–2187	2–3706	1–7643
Occipital WMH	0–7684	0–4028	6–941	1–2689	0–7684
Total WMH	402–137751	422–111993	635–25874	629–45093	402–137751

*Note*: Age, education, and BMI are reported as the mean ± SD, whereas sex, diagnostic status, hypertension, and diabetes are presented as the total number of participants and the percentage of the sample for each group. WMH, white matter hyperintensity load. Values are presented as the minimum and maximum raw WMH values for each group. Values are presented in mm^3^.

Abbreviations: AD, Alzheimer's disease; BMI, body mass index; MCI, mild cognitive impairment; MRI, magnetic resonance imaging; NC, normal control; SD, standard deviation; WMH, white matter hypointensity.

^a^ Represents statistically significant racial group differences. Black participants had lower education, lower proportions of male participants, higher rates of diabetes and hypertension, and higher BMIs.

^b^ Represents statistically significant racial group differences. Asian participants were older, had lower BMIs, and higher rates of diabetes and hypertension compared to White participants.

^c^ Represents statistically significant ethnic group differences. Hispanic participants had lower education levels, higher BMIs, and higher rates of diabetes and hypertension compared to non‐Hispanic participants.

The clinical dataset was used to examine both longitudinal and baseline WMH burden, whereas the MRI subset was used to examine only baseline WMH volume. Therefore, the MRI subset did not include repeated WMH measures. The MRI subset included a smaller sample comprising 2409 non‐Hispanic participants (1876 White, 260 Black, and 55 Asian) with completed race information. This subset also included 2398 participants (2191 non‐Hispanic and 207 Hispanic) with completed ethnicity information. Participant ages ranged from 50 to 95 years.

For each participant, cognitive diagnoses were determined using the NACCETPR and NACCTMCI variable names. Participants diagnosed with AD were identified through the NACCETPR variable, which represents the primary etiologic diagnosis. The variable “NACCETPR” represents the primary etiological diagnosis in the NACC dataset, categorizing participants into different clinical diagnoses such as no impairment, or AD. The “NACCTMCI” variable categorizes the type of MCI diagnosed in participants who do not have AD and do not exhibit normal cognition, according to clinician assessments. This variable includes classifications such as amnestic MCI, either single or multiple domain, and non‐amnestic MCI, also in single or multiple domains.

### 2.2 Vascular risk factors

Body mass index (BMI) was calculated using the NACCBMI column, which was provided based on height and weight measurements recorded during the visit. Hypertension was defined by merging values from HYPERT, HXHYPER, and HYPERTEN columns in NACC, with individuals without hypertension assigned a value of “0” and individuals with hypertension assigned value of “1”. Diabetes was defined using the values derived from columns DIABET and DIABETES. A value of “0” indicated no diabetes, whereas value of “1” was categorized as diabetes present.

### 2.3 Volumetric WMH measurements

T1w scans were pre‐processed through our standard pipeline including noise reduction,^37^ intensity inhomogeneity correction,^38^ and intensity normalization into range [0–100]. The pre‐processed images were linearly (nine parameters: three translation, three rotation, and three scaling)^39^ registered to the MNI‐ICBM152‐2009c average.^40^

A previously validated WMH segmentation technique was used to obtain WMH measurements.^41^ This technique has been employed previously in other multi‐center studies^42^, ^43^ as well as in NACC cohorts.^2^, ^42^ The automated WMH segmentation technique extracts a set of location (i.e., spatial priors) and intensity (distribution histograms) features and uses them in combination with a random forest classifier to detect the WMHs in new images.^38^, ^39^, ^40^ Automatic segmentation of the WMHs was completed using only the T1w contrasts, since NACC did not have FLAIR images available for all participants. We have previously validated the performance of our pipeline in detecting WMHs based on T1w images, and have shown that the T1w‐based WMH volumes hold strong correlations with FLAIR‐based WMH volumes (*r*  =  .97, *p*  < 0.001). Finally, the quality of all preprocessing steps and WMH segmentations was visually assessed (blinded to clinical diagnosis). WMH load was defined as the volume of all voxels identified as WMH in the standard space (in mm^3^) and was thus normalized for head size. Regional (frontal, temporal, occipital, and parietal) and total WMH volumes were calculated based on Hammers Atlas.^41^, ^44^ All WMH volumes were also log‐transformed to achieve normal distribution.

### 2.4 Statistical analysis

Group comparisons for vascular risk factors were conducted using independent samples *t*‐tests for continuous measures (BMI, age, and education) and chi‐square (*x* ^2^) tests for categorical measures (diabetes, hypertension, and sex ratios). To examine the influence of race and ethnicity on WMHs, we employed a combination of linear regression and linear mixed‐effects models. Baseline analyses utilized linear regression to assess whether race was associated with WMH burden, whereas longitudinal analyses used mixed‐effects models to account for repeated measures over time. WMH burden was defined using clinical categories (low, moderate, and extensive) derived from the IMAGMWMH and IMAGEWMH variables. Additional analyses were conducted separately for the total WMH measure and for regional WMH volumes (frontal, parietal, temporal, and occipital lobes) in the subset of participants with MRI data.

### 2.5 Baseline analysis

Baseline WMH was modeled as a function of race and adjusted for age, sex, education, and diagnosis. Diagnosis was included to control for potential differences due to diagnostic grouping between the races. Race was treated as a categorical variable, with comparisons conducted for Black versus White and White versus Asian. Ethnicity was also treated as a categorical variable comparing Hispanic to non‐Hispanic participants (equation 1).

(1) WMH∼Race/Ethnicity+Age+Sex+Education+Diagnosis

### 2.6 Longitudinal analysis

To assess WMH burden over time, linear mixed‐effects models were used, incorporating participant ID as a random effect to account for repeated measures in the same participant. This approach allowed us to model the effects of race and covariates on the longitudinal progression of WMH burden (equation 2).

(2) $$WMH∼Race/Ethnicity+Age+Sex+Education+Diagnosis+\left(1|ID\right)$$

### 2.7 Additional analyses controlling for vascular risk factors

To explore the contribution of vascular risk factors, additional models included diabetes, hypertension, and BMI as covariates. The analyses were performed for both baseline and longitudinal WMH to evaluate whether racial and ethnic differences persisted after accounting for vascular health disparities (equation 3).

(3) $$\begin{array}{ccc} WMH & ∼ & Race/Ethnicity+Age+Sex+Education+Diagnosis\\ & & +\;Diabetes+Hypertension+BMI \end{array}$$

For longitudinal analyses, the same model structure was extended by including participant ID as a random effect (equation 4):

(4) $$\begin{array}{ccc} WMH & ∼ & Race/Ethnicity+Age+Sex+Education+Diagnosis\\ & & +\;Diabetes+Hypertension+BMI+\left(1|ID\right) \end{array}$$

An additional analysis was conducted to determine if the rate of WMH progression differed across racial/ethnic groups by incorporating an interaction term between race and time from baseline, with and without controlling for vascular factors (equations 5 and 6).

(5) $$\begin{array}{ccc} WMH & ∼ & Race/Ethnicity+AgeBaseline+Sex+Education+Diagnosis\\ & & +\;TimeFromBaseline+Race:TimeFromBaseline+\left(1|ID\right) \end{array}$$

(6) $$\begin{array}{ccc} WMH & ∼ & Race/Ethnicity+AgeBaseline+Sex+Education+Diagnosis\\ & & +\;Diabetes+Hypertension+BMI+TimeFromBaseline\\ & & +\;Race/Ethnicity:TimeFromBaseline+\left(1|ID\right) \end{array}$$

To address imbalances (in age, sex, education levels, diagnostic group) across our groups of interest, a bootstrapping method was employed. This method involves repeatedly subsampling the larger subgroup (i.e., Whites or non‐Hispanics) to match the smaller subgroups (i.e., Blacks, Asians, and Hispanics) for these variables and calculating the indirect effect for each resampled subset.^45^ For each iteration, we randomly selected subsets of White participants to match the number of participants in the smaller racial and ethnic groups (i.e., Black, Hispanic, and Asian participants) based on age, sex, education, and diagnosis. For instance, to compare Black and White participants, 934 White participants were randomly resampled from the original pool of 7772 Whites to match the number of Black participants based on sex, education, diagnosis, and age. This process was repeated 1000 times, generating balanced samples for each racial and ethnic group comparison. The same approach was applied for Asians (288 participants) and Hispanics (636 participants), ensuring balanced group sizes and characteristics. This methodology aligns with best practices for handling unbalanced datasets in population‐level research, as described in prior studies.^25^, ^45^ The resampling process was applied to both the clinical dataset and the MRI subset.

### 2.8 Regional WMH sub‐analyses

A sub‐analysis was conducted to investigate global and regional WMH volumes (frontal, parietal, temporal, and occipital lobes) at baseline across Black versus White, White versus Asian, and Hispanic versus non‐Hispanic groups. WMHs for this sub‐analysis were derived from MRI data, focusing on regional and total WMHs for each racial and ethnic group. For this sub‐analysis, the baseline models (1 and 3) were repeated using the regional WMH measures. Similar to the clinical dataset, to manage group imbalance, a bootstrapping method was employed in the sub‐analysis with available MRI data. For example, White participants were resampled 264 times from the original clinical dataset of 2085 to get 1000 new datasets with balanced samples matching Black participants based on sex, education, diagnosis, and age. All analyses were conducted in MATLAB R2019b.

## 3 RESULTS

### 3.1 Follow‐up times between groups

The average number of repeated assessments and the time between assessments were generally similar across groups. Hispanic participants had slightly more repeated assessments (1.95 vs 1.85, *p *= 0.04) compared to non‐Hispanics, with a comparable mean time between assessments (45276 days vs 454.69 days, *p *= 0.76). However, non‐Hispanics exhibited greater variability in the range of time between assessments (6–2204 days vs 196–1474 days).

Regarding race, White participants had slightly more repeated assessments (1.87 vs 1.71, *p *< 0.01) compared to Black participants, with a similar mean time between visits (454.18 days v. 463.00 days, *p *= 0.27). Black participants had a slightly narrower range (203–1792 days) compared to White participants (6–2204 days). When comparing White to Asian participants, they had a ssimilar average number of repeated assessments (1.94 vs 1.87, *p *= 0.33) and mean time between visits (446.95 vs 454.18 days, *p *= 0.50), although Asians had a narrower range than White participants (245–1404 days vs 6–2204 days). These findings indicate small non‐significant differences in follow‐up assessments between the groups.

### 3.2 WMH progression over time

Our results indicate a significant interaction between WMH progression over time by race. Compared to Asians, both White (*t *= 2.48, *p *= 0.013) and Black participants (*t *= 3.08, *p *= 0.002), had increased rate of WMH progression over time. The White and Black participants did not differ in rate of change over time (*t *= 1.68, *p *= 0.093). This finding suggests that Asian individuals experience less WMH progression compared to White and Black individuals, who do not differ.

### 3.3 Vascular risk factors

Table 1 presents the demographic and descriptive information for vascular factors and diagnostic status across racial and ethnic groups for the subset with visual WMH assessments. Please see Table S1 in the supplementary section comparing the MRI sub‐sample to the overall clinical sample. White participants had significantly higher education levels than Black participants (*t *= 12.38, *p *< 0.001) but not Asian participants (*t *= 0.89, *p *= 0.37). Hispanic participants also had significantly lower education levels compared to non‐Hispanics (*t *= –14.39, *p *< 0.001). Regarding BMI, Black participants (*t *= 11.46, *p *< 0.001) had significantly higher BMIs than White participants, and Hispanic participants (*t *= 5.86, *p *< 0.001) had significantly higher BMIs than non‐Hispanic participants. Asian participants had significantly lower BMIs than White participants (*t *= –12.30, *p *< 0.001). There were no statistically significant age differences between White and Asian participants (*t *= ‐0.22, *p *= 0.82) or between Hispanic and non‐Hispanic participants (*t *= 0.25, *p *= 0.80), although Black participants were slightly older than White participants (*t *= 2.85, *p *= 0.004).

The chi‐square analyses revealed significant group differences in sex distribution. Whites had a higher proportion of male participants (47%) compared to Blacks (27%; χ^2 ^= 124.97, *p *< 0.001). Non‐Hispanics had a higher proportion of male participants (45%) compared to Hispanics (37%; ±^2 ^= 20.31, *p *< 0.001), and there was a statistically significant difference in sex distribution between Whites and Asians (*χ* ^2 ^= 3.92, *p *= 0.05). In addition, statistically significant group differences were observed for vascular risk factors. Black participants had significantly higher rates of diabetes (*χ* ^2 ^= 240.53, *p *< 0.001) and hypertension (*χ* ^2 ^= 245.11, *p *< 0.001) compared to White older adults. Similarly, Hispanic participants had higher rates of diabetes (*χ* ^2 ^= 106.65, *p *< 0.001) and hypertension (*χ* ^2 ^= 32.48, *p *< 0.001) than non‐Hispanics. Although White participants had higher rates of diabetes compared to Asian participants (*χ* ^2 ^= 21.94, *p *< 0.001), no statistically significant differences were observed for hypertension between White and Asian participants (*χ* ^2 ^= 1.98, *p *= 0.16).

Table 2 presents the demographic and descriptive information for vascular factors and diagnostic status across racial and ethnic groups in WMH sub‐analysis with available MRI data. White participants had significantly higher education than Black participants (*t *= 6.87, *p *< 0.001), whereas Black participants had higher BMIs than White participants (*t *= 8.27, *p *< 0.001). The chi‐square analysis revealed a significant difference in sex distribution between Whites and Blacks (*χ* ^2 ^= 27.65, *p *< 0.001), with a higher proportion of male participants in the White group. Significant racial group differences were also found for the proportion of participants with diabetes (*χ* ^2 ^= 141.56, *p *< 0.001) and hypertension (*χ* ^2 ^= 71.56, *p *< 0.001), with Black participants showing higher rates of both conditions than White participants.

Comparing Whites and Asians, White participants were younger (*t *= 3.33, *p *= 0.001) and had higher BMIs (*t *= 7.64, *p *< 0.001). No statistically significant differences were observed in education levels (*t *= −0.75, *p *= 0.45) or sex distribution (*χ* ^2 ^= 2.53, *p *= 0.11). However, a chi‐square analysis revealed a significant difference in the proportion of participants with diabetes (*χ* ^2^ = 13.15, *p *< 0.001) and hypertension (χ^2^ = 4.22, *p* = 0.04), with Asians participants having higher rates compared to White. In the comparison of Hispanic and non‐Hispanic participants, Hispanics were older (*t *= 3.08, *p *= 0.002) and had higher BMIs (*t *= 4.91, *p *< 0.001) but lower education levels (*t *= −13.06, *p *< 0.001) than non‐Hispanics. The chi‐square analysis indicated significant differences in sex distribution (*χ* ^2 ^= 10.79, *p *= 0.001), diabetes (*χ* ^2 ^= 39.18, *p *< 0.001), and hypertension (*χ* ^2 ^= 21.45, *p *< 0.001), with Hispanics showing higher rates of diabetes and hypertension compared to non‐Hispanics.

### 3.4 WMH burden differences without controlling for vascular risk factors

**FIGURE 1:**
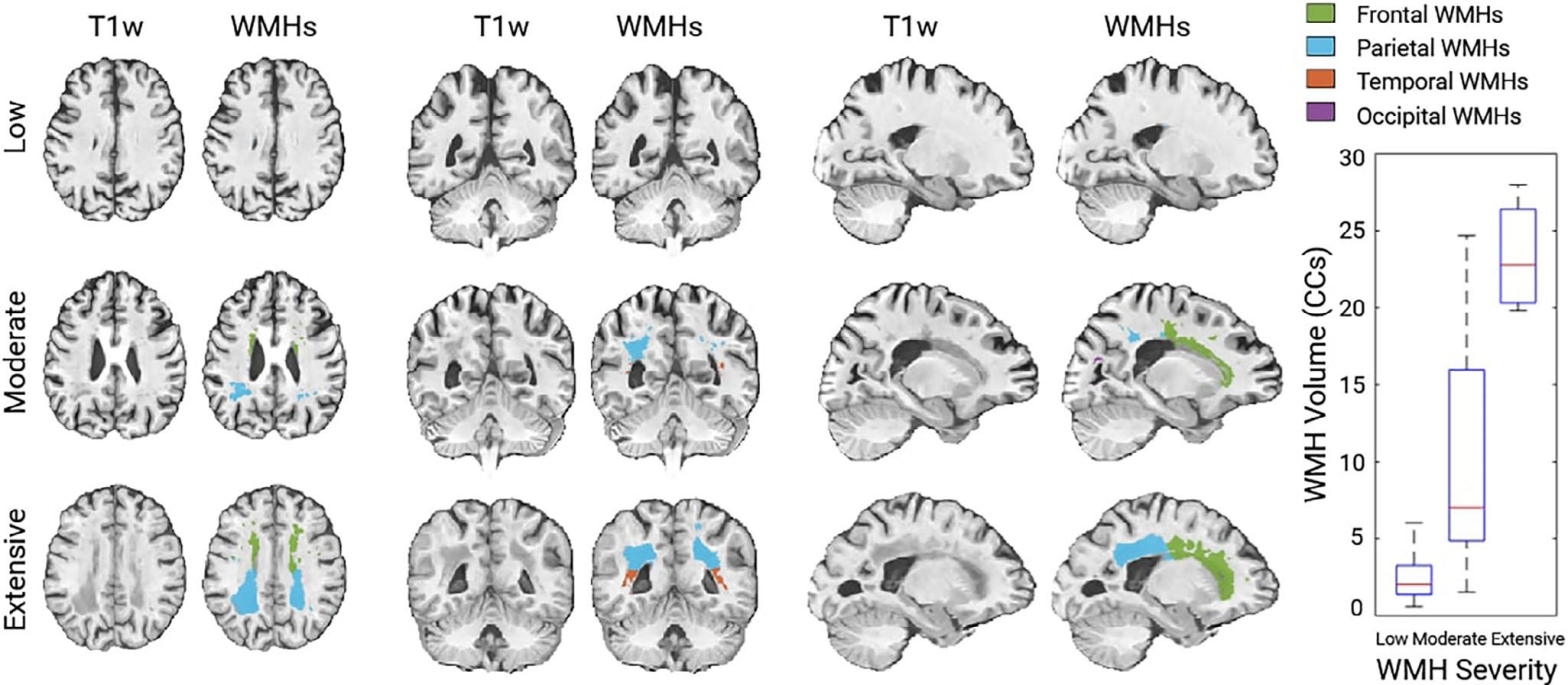
Examples of volumetric WMH segmentations for three NACC participants (age: 75 years) with low, moderate, and extensive WMH burden based on visual assessments. The boxplots show the distribution of volumetric WMHs (in cubic centimeters; CCs) for the subset of 292 participants that had both visual and volumetric WMH assessments available. NACC, National Alzheimer's Coordinating Center; WMH, white matter hyperintensity.

Table 3 presents cross‐sectional, longitudinal, and WMH progression analyses, comparing median effect sizes, t‐statistics, and confidence intervals (CIs) across racial and ethnic groups without controlling for vascular risk factors. Figure 1 provides an example of regional WMH segmentations for three, 75‐year‐old NACC participants with low, moderate, and extensive WMH burden (based on visual ratings). For cross‐sectional analyses, significant differences in WMH burden were observed between Black and White participants (median T‐stat = 4.77, *p* < 0.001), with 95% CIs ranging from 3.57 to 6.06, and 99.5% CIs ranging from 3.26 to 6.38. These findings indicate that Black individuals had a higher WMH burden compared to matched White individuals after controlling for age, sex, education, and diagnostic status. Conversely, no statistically significant group differences were observed between Asian and White participants (median T‐stat = 0.51, *p* = 0.65), with 95% CIs (–0.74 to 1.95) and 99.5% CIs (–1.16 to 2.50) including zero, suggesting no meaningful differences in WMH burden between these groups. Similarly, no statistically significant differences were observed between Hispanics and non‐Hispanics (median T‐stat = –1.45, *p* = 0.15), with 95% CIs (–2.76 to ‐0.20) and 99.5% CIs (–3.14 to 0.13).

**TABLE 3:** Confidence intervals for the t‐statistic across 1000 iterations, without controlling for vascular risk factors.

Model	Groups	Median effect size	Median T stat	Lower 99.5% CI	Upper 99.5% CI	Lower 95% CI	Upper 95% CI
Model 1	**Baseline**						
	Blacks vs Whites	0.16	4.77	3.26	6.38^a^	3.57	6.06^a^
	Asians vs Whites	0.03	0.51	−1.16	2.50	−0.74	1.95
	Hispanics vs non‐Hispanics	−0.05	−1.45	−3.14	0.13	−2.76	−0.20
Model 2	**Longitudinal**						
	Blacks vs Whites	0.12	5.41	4.10	6.71^a^	4.38	6.42^a^
	Asians vs Whites	0.03	0.87	−0.44	2.32	−0.14	2.00
	Hispanics vs non‐Hispanics	−0.04	−1.72	−2.76	−0.45	−2.50	−0.74
Model 5	***WMH progression across time***						
	Blacks vs Whites	0.12	5.51	4.19	6.85^a^	4.48	6.53^a^
	Asians vs Whites	0.02	0.71	−0.61	2.16	−0.28	1.86
	Hispanics vs non‐Hispanics	−0.04	−1.68	−2.73	−0.41	−2.46	−0.69

Abbreviations: CI, confidence interval; WMH, white matter hyperintensity.

^a^ t‐statistic is significant for group differences.

For longitudinal data, Black participants continued to exhibit higher WMH burden compared to White participants (median T‐stat = 5.41, *p* < 0.001), with 95% CIs (4.38 to 6.42) and 99.5% CIs (4.10 to 6.71), highlighting robust and persistent differences. Asian and White participants again showed no statistically significant differences in WMH progression over time (median T‐stat = 0.87, *p* = 0.58), with 95% CIs (–0.14 to 2.00) and 99.5% CIs (–0.44 to 2.32). Similarly, no statistically significant differences were observed for Hispanics compared to non‐Hispanics (median T‐stat = –1.72, *p* = 0.08), with 95% CIs (–2.50 to –0.74) and 99.5% CIs (–2.76 to –0.45).

When examining the rate of WMH progression across time, significant differences persisted between Black and White participants (median T‐stat = 5.51, *p* < 0.001), with 95% CIs (4.48 to 6.53) and 99.5% CIs (4.19 to 6.85). Asian and White participants showed no significant differences (median T‐stat = 0.71, *p* = 0.15), with 95% CIs (–0.28 to 1.86) and 99.5% CIs (–0.61 to 2.16). Hispanics and non‐Hispanics did not differ significantly in progression rates (median T‐stat = –1.68, *p* = 0.09), with 95% CIs (–2.46 to –0.69) and 99.5% CIs (–2.73 to –0.41).

### 3.5 WMH burden differences after controlling for vascular risk factors

Table 4 presents the results of models controlling for vascular risk factors, providing median effect sizes, t‐statistics, and CIs for cross‐sectional, longitudinal, and WMH progression analyses. When vascular risk factors were included in the models, cross‐sectional differences in WMH burden between Black and White participants remained statistically significant (median T‐stat = 3.14, *p* < 0.001), although the effect was attenuated compared to models not controlling for vascular risk factors (95% CIs = 1.92 to 4.47; 99.5% CIs = 1.53 to 4.95), suggesting that vascular risk factors account for some of the observed group differences. No statistically significant differences were observed between Asian and White participants (median T‐stat = 0.66, *p* = 0.59), with 95% CIs (–0.75 to 2.13) and 99.5% CIs (–1.14 to 2.57), or between Hispanic and non‐Hispanic participants (median T‐stat = –1.73, *p* = 0.07), with 95% CIs (–2.98 to –0.53) and 99.5% CIs (–3.37 to –0.03).

**TABLE 4:** Confidence intervals for the t‐statistic across the 1000 iterations, controlling for vascular risk factors.

Model	Groups	Median effect size	Median T stat	Lower 99.5% CI	Upper 99.5% CI	Lower 95% CI	Upper 95% CI
Model 3	**Baseline**						
	Blacks vs Whites	0.10	3.14	1.53	4.95^a^	1.92	4.47^a^
	Asians vs Whites	0.04	0.66	−1.14	2.57	−0.75	2.13
	Hispanics vs non‐Hispanics	−0.07	−1.73	−3.37	−0.03	−2.98	−0.53
	**Longitudinal**						
Model 4	Blacks vs Whites	0.09	4.43	3.06	5.78^a^	3.37	5.45^a^
	Asians vs Whites	0.03	0.81	−0.72	2.42	−0.33	2.00
	Hispanics vs non‐Hispanics	−0.05	−2.00	−3.04	−0.77^a^	−2.81	−1.02^a^
	***WMH progression across time***						
Model 6	Blacks vs Whites	0.09	4.55	3.18	5.59^a^	3.48	5.59^a^
	Asians vs Whites	0.02	0.66	−0.87	2.27	−0.49	1.85
	Hispanics vs non‐Hispanics	−0.05	−1.96	−3.05	−0.73^a^	−2.77	−0.96^a^

Abbreviations: CI, confidence interval; WMH, white matter hyperintensity.

^a^ t‐statistic is significant for group differences.

In longitudinal data, WMH burden for Black participants compared to White participants remained significant (median T‐stat = 4.43, *p* < 0.001), although the effect was attenuated relative to the model excluding vascular risk factors (95% CIs = 3.37 to 5.45; 99.5% CIs = 3.06 to 5.78). Differences between Asian and White participants remained non‐significant (median T‐stat = 0.81, *p* = 0.59), with 95% CIs (–0.33 to 2.00) and 99.5% CIs (–0.72 to 2.42). However, differences between Hispanic and non‐Hispanic participants were significant (median T‐stat = –2.00, *p* = 0.04), with 95% CIs (–2.81 to –1.02) and 99.5% CIs (–3.04 to –0.77).

**FIGURE 2:**
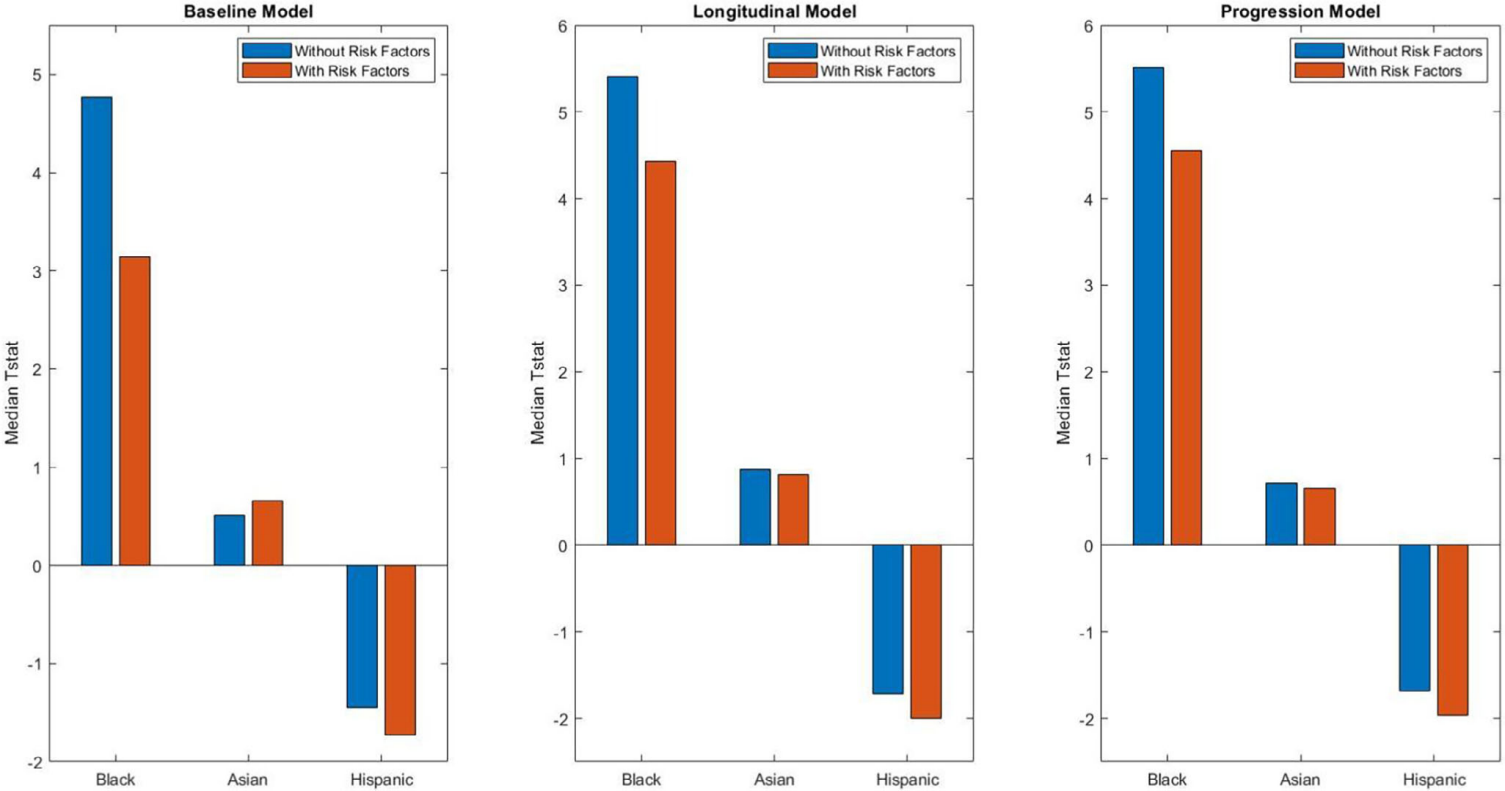
Illustrates the median t‐statistics results from 1000 linear regressions assessing the effect of race (Black or Asian vs White) and ethnicity (Hispanic vs non‐Hispanic) on total WMH burden and progression in the clinical dataset. All models adjust for age, sex, education, and diagnostic status. “With Risk Factors” models additionally control for vascular risk factors (BMI, hypertension, and diabetes). BMI, body mass index; WMH, white matter hypointensity.

For the rate of WMH progression across time, significant differences persisted between Black and White participants (median T‐stat = 4.55, *p* < 0.001), although the strength of association was reduced (95% CIs = 3.48 to 5.59; 99.5% CIs = 3.18 to 5.59). Asian and White participants showed no significant differences in WMH progression (median T‐stat = 0.66, *p* = 0.17), with 95% CIs (–0.49 to 1.85) and 99.5% CIs (‐0.87 to 2.27). However, Hispanics and non‐Hispanics exhibited significant differences across time (median T‐stat = –1.96, *p* = 0.04), with 95% CIs (–2.77 to –0.96) and 99.5% CIs (–3.05 to –0.73) (see Figure 2).

To assess whether the rate of WMH progression varies by baseline age, we extended our longitudinal models to include a time*age (at baseline) interaction term. The results showed no change in model performance or explanatory power, indicating that age as a linear covariate sufficiently accounts for age‐related differences in WMH progression.

### 3.6 MRI dataset: Impact of clinical syndrome

An additional analysis was done to examine the main effect of diagnosis on WMH volume. Overall, MCI and AD participants had more WMH volume compared to healthy older adults/normal controls (NCs). Those with MCI and AD exhibited increased WMH compared to NCs for total, frontal, temporal, parietal, and occipital regions (*t* belongs to [5.23–3.19], *p *< 0.05). No statistically significant differences were observed between MCI and AD. These results suggest that although WMH volume is significantly elevated in cognitive impairment compared to healthy adults, differences between MCI and AD are minimal across all regions.

We also analyzed the effect of diagnosis on WMH volume by race and ethnicity separately. Non‐Hispanic MCI and AD participants exhibited significantly greater WMH volume than NCs in total, frontal, parietal, and temporal regions (*t* belongs to [4.97–2.82], *p* < 0.05). For White participants, MCI and AD participants had significantly greater WMH volume than NCs in total, frontal, parietal, temporal, and occipital regions (*t* belongs to [5.31–2.73], *p* < 0.05). No statistically significant differences were found between MCI and AD across any region for any race and ethnicity except Blacks, who had slightly greater WMH volume in AD compared to MCI only in the temporal region (*t *= –2.00, *p* = 0.046).

### 3.7 MRI dataset: WMH volume differences without controlling for vascular risk factors

Table 5 presents the regional and total WMH volume in the MRI dataset, with and without controlling for vascular risk factors. Significant differences in total WMH volume were observed between Black and White participants (median T‐stat = 5.29, *p* < 0.001), with 95% CIs ranging from 3.97 to 6.57, and 99.5% CIs from 3.46 to 6.92. The regional analysis revealed significantly higher WMH volume in Black participants compared to White participants in all regions examined. Frontal WMH differences were notable (median T‐stat = 4.91, *p* < 0.001), with 95% CIs (3.59 to 6.16) and 99.5% CIs (3.21 to 6.44). Parietal WMHs showed the strongest effect size among regions (median T‐stat = 4.92, *p* < 0.001), with 95% CIs (3.68 to 6.20) and 99.5% CIs (3.28 to 6.61). Temporal WMH volume was also significantly higher in Black participants (median T‐stat = 2.07, *p* = 0.03), with 95% CIs (0.92 to 3.29) and 99.5% CIs (0.48 to 3.74). Occipital WMH exhibited a statistically significant difference (median T‐stat = 4.64, *p* < 0.001), with 95% CIs (3.52 to 5.85) and 99.5% CIs (3.20 to 6.44).

**TABLE 5:** Confidence intervals for the t‐statistic across the 1000 iterations in the MRI dataset comparing Black and White older adults.

Model		Median effect size	Median T stat	Lower 99.5% CI	Upper 99.5% CI	Lower 95% CI	Upper 95% CI
Model 1	*Vascular risk factors not included*						
	Total WMH	0.33	5.29	3.46	6.92	3.97	6.57^a^
	Frontal WMH	0.3	4.91	3.21	6.44	3.59	6.16^a^
	Parietal WMH	0.31	4.92	3.28	6.61	3.68	6.20^a^
	Temporal WMH	0.13	2.07	0.48	3.74	0.92	3.29^a^
	Occipital WMH	0.29	4.64	3.2	6.44	3.52	5.85^a^
Model 3	*Vascular risk factors included*						
	Total WMH	0.25	4.07	2.2	5.62	2.67	5.33^a^
	Frontal WMH	0.22	3.58	2.05	5.13	2.3	4.75^a^
	Parietal WMH	0.22	3.61	1.88	5.19	2.33	4.79^a^
	Temporal WMH	0.11	1.76	0.11	3.4	0.53	3.06
	Occipital WMH	0.27	4.37	2.98	5.99	3.2	5.63^a^

Abbreviations: CI, confidence interval; WMH, white matter hyperintensity.

^a^ t‐statistic is significant for group differences.

In contrast, no statistically significant differences in WMH volume were observed between Asian and White participants across all regions (all *p*’s > 0.05), including total WMH (median T‐stat = 1.00, *p* = 0.31, 95% CIs = –0.54 to 2.67, 99.5% CIs = –1.10 to 3.09). Similarly, no significant differences in WMH volume were observed between Hispanic and Non‐Hispanic participants across all regions, including total WMH (median T‐stat = 1.04, *p* = 0.39, 95% CIs = –0.20 to 2.16, 99.5% CIs = –0.68 to 2.52). Regional analysis showed no statistically significant differences in frontal, parietal, temporal, or occipital WMH volume (all *p*’s > 0.05). The CIs for these comparisons consistently included zero, indicating no meaningful differences. Please see Tables S2 and S3 in the supplementary section for additional details on CIs for the t‐statistic comparing Asian and White older adults and Hispanic compared to non‐Hispanic older adults.

### 3.8 MRI dataset: WMH volume differences after controlling for vascular risk factors

**FIGURE 3:**
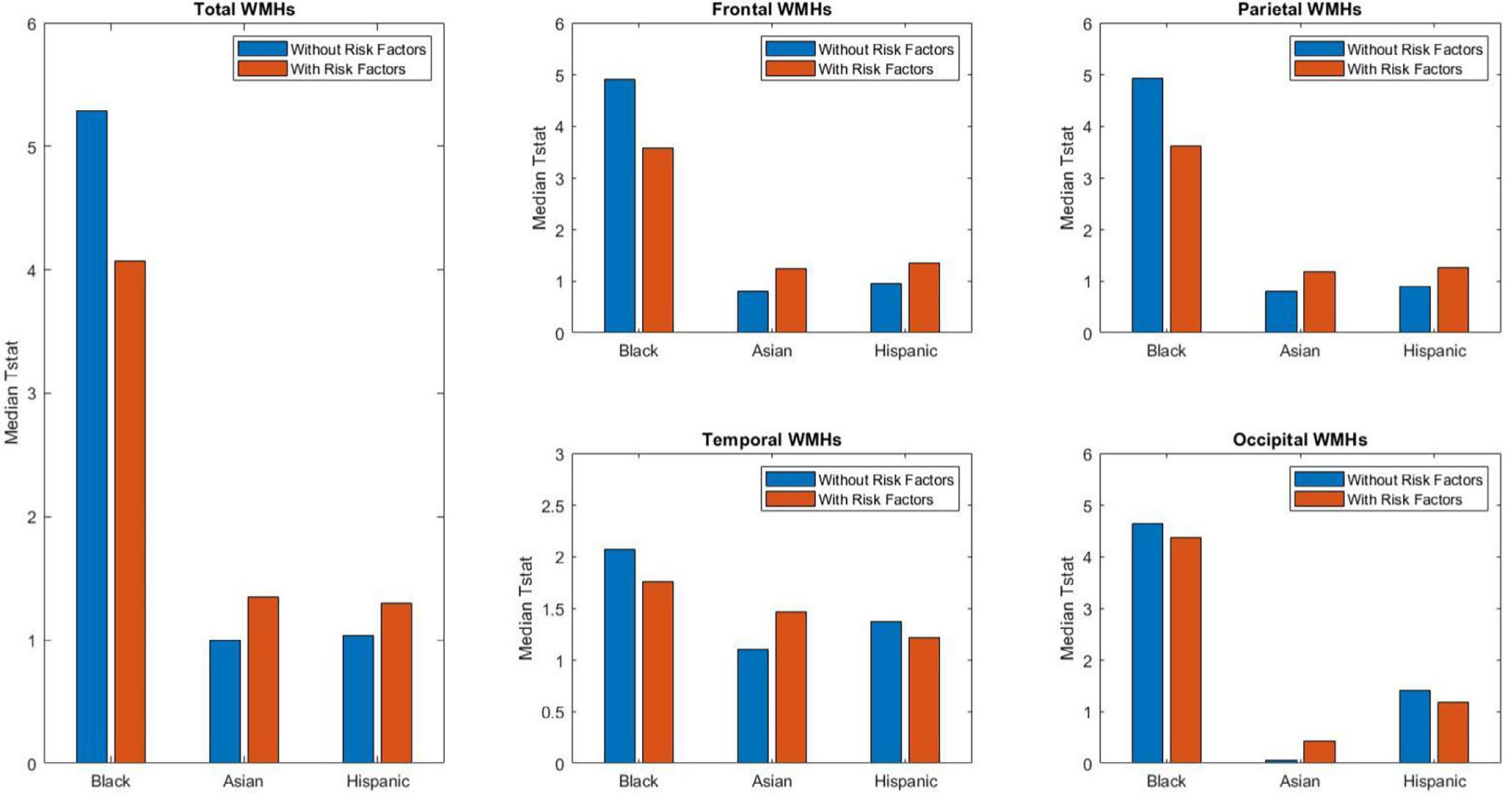
Illustrates the median t‐statistics results from 1000 linear regressions assessing the effect of race (Black or Asian vs White) and ethnicity (Hispanic vs non‐Hispanic) on volumetric total and regional WMH volume in the MRI dataset. All models adjust for age, sex, education, and diagnostic status. “With Risk Factors” models additionally control for vascular risk factors (BMI, hypertension, and diabetes). BMI, body mass index; MRI, magnetic resonance imaging; WMH, white matter hypointensity.

When vascular risk factors were included in the models, the differences in total WMH volume between Black and White participants remained significant (median T‐stat = 4.07, *p* < 0.001), although the effect size and CIs were much reduced (95% CIs = 2.67 to 5.33, 99.5% CIs = 2.20 to 5.62) (see Figure 3). Regional analysis showed that frontal WMH volume remained significantly elevated in Black participants (median T‐stat = 3.58, *p* < 0.001), with 95% CIs (2.30 to 4.75) and 99.5% CIs (2.05 to 5.13). Although the effect size was smaller when vascular risk factors were added, parietal WMH differences were still statistically significant (median T‐stat = 3.61, *p* < 0.001), with 95% CIs (2.33 to 4.79) and 99.5% CIs (1.88 to 5.19). When vascular risk factors were included in the models, the differences in temporal WMH became non‐significant (median T‐stat = 1.76, *p* = 0.08), with 95% CIs (0.53 to 3.06) and 99.5% CIs (0.11 to 3.40). Occipital WMH volume between Black and White participants remained significant (median T‐stat = 4.37, *p* < 0.001), with 95% CIs (3.20 to 5.63) and 99.5% CIs (2.98 to 5.99).

No statistically significant differences in WMH volume were observed between Asian and White participants across all regions (all *p*’s > 0.05), including total WMH (median T‐stat = 1.35, *p* = 0.18, 95% CIs = –0.26 to 3.06, 99.5% CIs = –0.74 to 3.43). Although the effect size and CIs were larger when vascular risk factors were included, no significant differences in WMH volume were observed between Hispanic and Non‐Hispanic participants across all regions (all *p*’s > 0.05), including total WMH (median T‐stat = 1.30, *p* = 0.19, 95% CIs = 0.11 to 2.46, 99.5% CIs = –0.42 to 2.79).

## 4 DISCUSSION

This study investigated racial and ethnic differences in WMHs and vascular risk factors in older adults, using a large and diverse dataset from NACC. When using WMH severity staging (low, moderate, extensive – clinical dataset), for baseline and longitudinal measures, Black participants exhibited more WMH burden than White participants; however, this effect was attenuated after controlling for vascular risk factors. Similarly, when examining WMH volume (from the MRI dataset), regional Black–White racial differences were attenuated after controlling for vascular risk factors, with differences in the temporal region no longer significant. With respect to Hispanic versus non‐Hispanic individuals, there were no differences when examining baseline or longitudinal changes using the severity staging method when vascular risk factors were not controlled for. However, when adding vascular risk factors as covariates, longitudinal main effects and progression of WMH burden differences appeared between these groups. There were no differences between White and Asian older adults in terms of WMH burden or progression.

When examining both severity staging as well as volumetric measurements of WMH, the Black–White racial group differences were attenuated when controlling for vascular risk factors. In addition, temporal WMHs were no longer significant after controlling for vascular risk factors, aligning with previous research that suggest that racial differences in WMH volume are influenced by vascular risk factors.^25^, ^26^ These findings indicate that perhaps vascular risk factors are contributing to vascular pathology in AD, and controlling for these factors may reduce the disproportionate dementia risk in Black older adults. Other factors may also contribute to the increased burden, including smoking or alcohol use,^46^, ^47^ as well as chronic stress and experiences of racial discrimination, which have been implicated in accelerated biological aging and increased cerebrovascular damage.^16^ These psychosocial stressors can lead to dysregulation of the hypothalamic–pituitary–adrenal axis and heightened inflammatory responses, potentially promoting WMH accumulation independently of traditional vascular risk factors.^48^, ^49^ Socioeconomic factors such as lower access to health care, health literacy challenges, and environmental exposures may also play significant roles in the observed racial group WMH differences.^17^, ^50^ Future research is needed to explore these factors and their role in WMH burden.

Our findings reveal significant ethnoracial differences in WMH burden, particularly for White compared to Black and Hispanic groups, suggesting mechanisms that could lead to different cognitive health outcomes across races and ethnicity. Higher WMH volumes have been associated with greater disruptions in cerebral white matter integrity.^51^ For instance, tract‐specific white matter lesions correlate significantly with impairment in specific cognitive domains.^51^ These lesions compromise the structural connectivity within the brain, leading to inefficiencies in neural communication and subsequent cognitive declines, particularly in processing speed and executive functions. Moreover, regional differences observed in our study align with findings that specific brain areas, when affected by WMHs, can differentially impact cognitive capacities. Increased WMH volumes, especially in the frontal and temporal regions, are associated with more rapid cortical thinning and impact executive function and memory.^52^ These results highlight an important association between cognition, WMH, and brain structural degradation. Exploring regional WMHs can enhance our understanding of the neurobiological pathways through which racial and ethnic differences in cognitive health may arise.

For Hispanic participants, the findings present a unique and somewhat unexpected pattern. Baseline comparisons revealed no significant WMH differences compared to non‐Hispanic participants. However, after controlling for vascular risk factors, significant differences emerged in longitudinal WMHs and progression of WMHs. This finding could suggest that vascular risk factors are less predictive of WMH burden in Hispanic populations compared to non‐Hispanic groups, possibly due to differences in vascular risk factor thresholds or may suggest that other mechanisms contribute to ethnicity differences in WMH burden such as interactions with genetic, cultural, or environmental factors.^53^ These findings could also indicate that vascular factors mask the effects in this context,^54^ as significant differences emerged only when these factors were included in the model. A recent review highlights the complex interplay of systemic and cultural factors that shape cardiovascular outcomes in Hispanic populations, including the phenomenon known as the Hispanic paradox.^55^ This paradox describes how Hispanic individuals often exhibit better or comparable health outcomes despite higher rates of cardiovascular risk factors.^55^ Despite higher vascular risk factors, Hispanic populations did not exhibit increased WMH burden compared to non‐Hispanic populations, consistent with the Hispanic paradox. Systemic factors such as health care access, diet, and acculturation have been shown to influence the prevalence and impact of vascular risk factors in Hispanic populations differently than in Black or White populations,^53^, ^54^, ^56^ and therefore may also contribute to racial group differences in WMH burden. These factors may alter the predictive value of vascular risk factors and highlight the need for tailored approaches when examining ethnic disparities. Future studies should consider incorporating additional non‐vascular contributors, such as chronic stress, access to preventative care, and environmental exposures, to clarify these patterns and differences.

Despite the limited overlap between participants in clinical and MRI datasets (1146 participants had both WMH volume in MRI and WMH severity staging in the clinical dataset), the results for Black and Asian participants were consistent across the two datasets. However, this was not the case for Hispanic participants, where differences in cohort characteristics between the two datasets may explain the differences in the findings. The Hispanic cohort in the MRI dataset was older, less educated, and had higher BMI, hypertension, and diabetes rates, and included a greater proportion of cognitively normal participants compared to the clinical dataset. An additional analysis was thus carried out for Hispanic participants using the subset of the participants who had both WMH severity and volume measures. The results showed consistent negative t‐statistics values for Hispanics across both measures, suggesting that cohort differences, particularly in demographic and health characteristics, may have further contributed to the Hispanic differences in WMHs between the clinical and MRI datasets.

The examination of regional WMHs with vascular risk factors is particularly important because WMH volume in different brain regions is associated with distinct underlying causes. Previous research has shown that anterior WMH volume is strongly related to vascular risk factors.^29^, ^30^ Our results follow this trend as a larger reduction in WMH volume was observed in the frontal than occipital region when controlling for vascular risk factors. Regional measurements are thus important for understanding the specific contributions of vascular risk factors to overall WMH volume. Unfortunately, the regional dataset in the current study did not have longitudinal follow‐ups, which may explain why we did not observe differences between Hispanic and non‐Hispanic individuals.

There are some limitations in this study that need to be considered. First, the use of the NACC dataset, while providing a large and diverse sample, may introduce selection bias. Participants in the NACC are volunteers, and may be more educated and healthier than the general population, which could limit the generalizability of our findings.^57^ Another potential limitation is the use of the qualitative measures of WMH burden. This measurement method may offer lower precision than the MRI‐derived WMH volumes; however, the clinical dataset provides broader generalizability and statistical power due to its large sample size, complementing the smaller MRI subset. Third, although we included important vascular risk factors such as hypertension, diabetes, and BMI, other factors such as cholesterol, smoking, and physical inactivity, which can influence WMHs, were not included due to limited availability. In addition, the sample size for Asian older adults was relatively small (*n* = 55 in the MRI dataset), limiting the statistical power to detect significant differences in this group. The racial and ethnic composition of the NACC dataset reflects known differences in research participation, with underrepresentation of non‐White populations. Although bootstrapping was used to balance group comparisons, these findings should be interpreted with caution, as smaller sample sizes and potential sample biases for Black, Asian, and Hispanic groups may limit the generalizability of the results. Future research should prioritize recruitment of diverse populations to address this limitation. In addition, heterogeneity in acquisition protocols across the NACC dataset may contribute to variability in WMH quantification. Future studies should aim to assess and report potential systemic differences in imaging protocols across racial and ethnic groups to ensure robust comparisons. Similar to prior work in the literature, our analysis aggregated participants into broad racial and ethnic categories to ensure sufficient statistical power. Although this approach facilitated feasible comparisons, it may overlook heterogeneity within these groups, particularly among Asian and Hispanic/Latino participants. Future research should aim to recruit larger, more representative samples to allow for finer stratification, and investigation of within‐group differences.

Overall, we observed that vascular risk factors contribute to racial WMH burden differences between Black and White older adults. The increased WMH burden in Blacks is associated with increased rates of vascular risk factors, which may be a contributing factor to their increased risk of dementia compared to Whites. The reverse effect was observed in Hispanic versus non‐Hispanic populations, with vascular risk factors revealing ethnic group differences. These results highlight the importance of further exploring cultural, genetic, and environmental influences that may interact with vascular factors. Future studies should aim to include more comprehensive measures of vascular risk factors, such as cholesterol levels and glucose, alongside psychosocial factors such as discrimination, acculturation stress, and resilience.

## AUTHOR CONTRIBUTIONS

Farooq Kamal, Roqaie Moqadam, Mahsa Dadar, and Cassandra Morrison were involved with the conceptualization and design of the work. Farooq Kamal and Mahsa Dadar completed analysis and Cassandra Morrison, Mahsa Dadar, Roqaie Moqadam, and Farooq Kamal were involved with data interpretation. Farooq Kamal organized figures. Farooq Kamal wrote the manuscript, and Cassandra Morrison, Roqaie Moqadam, Mahsa Dadar, and Farooq Kamal revised and approved the submitted version.

## CONFLICT OF INTEREST STATEMENT

The authors declare no competing interests. Author disclosures are available in the Supporting Information.

## CONSENT STATEMENT

Written informed consent was obtained from participants or their study partners.

## DISCLOSURES

The authors report no disclosures relevant to the manuscript.

## Supporting information



Supporting Information

Supporting Information

## Data Availability

Data used in preparation of this article were also obtained from the National Alzheimer's Coordinating Center (NACC; https://naccdata.org/) database, including the NACC Uniform Data Set (UDS), and MRI Data Set (Beekly et al., 2004; Besser, Kukull, Knopman, et al., 2018; Besser, Kukull, Teylan, et al., 2018).
