# Supplementary material for: Racial and ethnic differences in white matter hypointensities: The role of vascular risk factors
Source: Alzheimers Dement. 2025 Mar 27;21(3):e70105. doi: 10.1002/alz.70105 (PMC11947760; doi:10.1002/alz.70105)
Supplement: Supplementary file 1 — Supporting Information [file ALZ-21-e70105-s001.docx]

**Supplementary Table 1.** Descriptive information for vascular risk factors in the clinical and MRI dataset

|  | **Clinical dataset (n=8307)** | **MRI dataset (n=2191)** |
| --- | --- | --- |
| Age (Mean ± SD) | 71.45 ± 9.20 | 70.55 ± 9.10 * |
| Education (Mean ± SD) | 16.18 ± 2.72 | 15.75 ± 2.92 * |
| Male (n, %) | 3724 (45%) | 963 (44%) |
| Diagnosis (%)     NC     MCI     AD | 4585 (55%)  779 (9%)  2943 (35%) | 1309 (60%) *  221 (10%)  661 (30%) * |
| Race/Ethnicity       White       Black       Asian       Hispanic | 7132 (86%)  892 (11%)  283 (3.4%)  661 | 1876 (86%)  260 (12%)  55 (2.5%) *  207 |
| BMI (Mean ± SD) | 26.88 ± 5.16 | 27.04 ± 4.89 |
| Hypertension (n, %) | 3746 (45%) | 991 (45%) |
| Diabetes (n, %) | 954 (12%) | 288 (13%) |

*Notes:* Age, education, and BMI are reported as the mean ± standard deviation, while sex, diagnostic status, hypertension, and diabetes are presented as the total number of participants and the percentage of the sample for each dataset.

*: Represents statistically significant dataset group differences. The MRI dataset had younger participants, had significantly lower education levels, a lower proportion of NC and AD, and a lower proportion of Asians compared to Clinical Dataset.

**Supplementary Table 2.** Confidence intervals for the t-statistic across the 1,000 iterations in the MRI dataset comparing Asian and White older adults

| Model |  | Median Effect Size | Median T stat | Lower 99.5% CI | Upper 99.5% CI | Lower 95% CI | Upper 95% CI |
| --- | --- | --- | --- | --- | --- | --- | --- |
|  | *Vascular Risk Factors not included* | |  |  |  |  |  |
| Model 1 | Total WMH | 0.14 | 1.00 | -1.10 | 3.09 | -0.54 | 2.67 |
|  | Frontal WMH | 0.11 | 0.81 | -1.28 | 3.02 | -0.71 | 2.50 |
|  | Parietal WMH | 0.11 | 0.81 | -1.41 | 3.10 | -0.81 | 2.41 |
|  | Temporal WMH | 0.15 | 1.11 | -1.00 | 2.94 | -0.37 | 2.63 |
|  | Occipital WMH | 0.01 | 0.07 | -1.93 | 2.23 | -1.38 | 1.57 |
|  | *Vascular Risk Factors included* | |  |  |  |  |  |
| Model 2 | Total WMH | 0.18 | 1.35 | -0.74 | 3.43 | -0.26 | 3.06 |
|  | Frontal WMH | 0.17 | 1.24 | -0.89 | 3.35 | -0.35 | 2.87 |
|  | Parietal WMH | 0.16 | 1.19 | -0.91 | 3.45 | -0.42 | 2.84 |
|  | Temporal WMH | 0.20 | 1.47 | -0.62 | 3.61 | -0.14 | 3.08 |
|  | Occipital WMH | 0.06 | 0.43 | -1.60 | 2.67 | -1.11 | 2.15 |

**Supplementary Table 3.** Confidence intervals for the t-statistic across the 1,000 iterations in the MRI dataset comparing Hispanic and Non-Hispanic older adults

| Model |  | Median Effect Size | Median T stat | Lower 99.5% CI | Upper 99.5% CI | Lower 95% CI | Upper 95% CI |
| --- | --- | --- | --- | --- | --- | --- | --- |
|  | *Vascular Risk Factors not included* | |  |  |  |  |  |
| Model 1 | Total WMH | 0.07 | 1.04 | -0.68 | 2.52 | -0.20 | 2.16 |
|  | Frontal WMH | 0.07 | 0.96 | -0.57 | 2.49 | -0.20 | 2.08 |
|  | Parietal WMH | 0.06 | 0.91 | -0.74 | 2.45 | -0.24 | 2.11 |
|  | Temporal WMH | 0.10 | 1.37 | -0.22 | 2.73 | 0.24 | 2.48 |
|  | Occipital WMH | 0.10 | 1.41 | -0.33 | 3.06 | 0.17 | 2.57 |
|  | *Vascular Risk Factors included* | |  |  |  |  |  |
| Model 2 | Total WMH | 0.09 | 1.30 | -0.42 | 2.79 | 0.11 | 2.46 |
|  | Frontal WMH | 0.10 | 1.35 | -0.26 | 2.87 | 0.14 | 2.41 |
|  | Parietal WMH | 0.09 | 1.27 | -0.38 | 2.89 | 0.12 | 2.43 |
|  | Temporal WMH | 0.09 | 1.22 | -0.35 | 2.67 | 0.10 | 2.33 |
|  | Occipital WMH | 0.08 | 1.18 | -0.67 | 2.85 | -0.06 | 2.30 |
|  |  |  |  |  |  |  |  |
